# Supplementary material for: Overt and occult hepatitis B virus infection detected among chronic kidney disease patients on haemodialysis at a Tertiary Hospital in Ghana
Source: PLoS One. 2024 Mar 4;19(3):e0290917. doi: 10.1371/journal.pone.0290917 (PMC10911607; doi:10.1371/journal.pone.0290917)
Supplement: Attachment — Submitted filename: Plos Reviewed.docx [file pone.0290917.s002.docx]

**Manuscript Number:** PONE-D-23-25809
**Manuscript Title:** Overt and occult hepatitis B virus infection detected among chronic kidney disease patients on haemodialysis at a Tertiary Hospital in Ghana

**The title and abstract cover the main aspect of the work?**

- Both the title and the abstract full     study. But the results of the        
- Results reported have not been published elsewhere.

**The introduction provide background and information relevant to the study?**

Some data and definitions have to be updated and well described.

- I am sending some critics and reference that could be interesting for the authors.
- A new classification of chronic hepatitis B natural history is well described in different publications
- Therapeutic strategies for hepatitis B virus infection: towards a cure Fannning et al,
- Clinical utility of HBV surface antigen quantification in HBV e antigen-negative chronic HBV infection. *Liaw Y-F.*
- The significance of isolated anti-HBc is not well described in this manuscript. Isolated Anti-HBc: Significance and Management. *Florian Moretto et al*
- The risk of HBV transmission by blood transfusion of anti-HBc positive units has to be discussed.
-         

**Are the methods clear and replicable? Do all the results presented match the methods described?**

- Yes, though the data analysis section could have used more details. For example, Was HBV infection defined as HCV DNA positivity or SAg positivity? Many of these details are unclear to me and so the data analysis section should be improved upon to clarify.
- The sensitivity and the specificity of the used kit should be mentioned since there is variability among the diagnostic test kits
- How can you define false Vs true occult hepatitis? You have to define Individuals with isolated anti-HBc-positive status viral loads of less/greater than number of copies/ml or IU/mL

**          **                         

Do the conclusions are presented in an appropriate fashion and are supported by the data? **No**. You mentioned in your conclusions like: The HBsAg-negative patients undergoing HD at the Renal Unit of KBTH was 7.7 % and 7.3%, respectively. Only 40.6% of the study subjects and 50.7% of the vaccinated patients were HBsAb-positive. This is not conclusion, but direct copy of the results. Please try to present your conclusions in an appropriate fashion and are supported by the data reported.

Does the article is presented in an intelligible fashion and is written in Standard English? No, the manuscript requires English editing to correct the grammar or flow

Does the research meets all applicable standards for the ethics of experimentation and research integrity? Yes

Does the article adheres to appropriate reporting guidelines and community standards for data availability? Yes
