# Supplementary material for: Overt and occult hepatitis B virus infection detected among chronic kidney disease patients on haemodialysis at a Tertiary Hospital in Ghana
Source: PLoS One. 2024 Mar 4;19(3):e0290917. doi: 10.1371/journal.pone.0290917 (PMC10911607; doi:10.1371/journal.pone.0290917)
Supplement: Attachment — Submitted filename: Response to Reviewers_PONE-D-23-25809.docx [file pone.0290917.s003.docx]

***Overt and occult hepatitis B virus infection detected among chronic kidney disease patients on haemodialysis at a Tertiary Hospital in Ghana***

**Response to Journal’s requirements:**

1. Please ensure that your manuscript meets PLOS ONE's style requirements, including those for file naming. The PLOS ONE style templates can be found at

Response: The revised manuscript has been formatted in accordance with PLOS ONE’s style requirements.

2. We suggest you thoroughly copyedit your manuscript for language usage, spelling, and grammar. If you do not know anyone who can help you do this, you may wish to consider employing a professional scientific editing service. Upon resubmission, please provide the following:

The name of the colleague or the details of the professional service that edited your manuscript

Response: All the authors have thoroughly reviewed and edited the revised manuscript for language usage, spelling and grammar.

A copy of your manuscript showing your changes by either highlighting them or using track changes (uploaded as a *supporting information* file)

Response: A copy of the revised manuscript showing the changes (using track changes) has been uploaded as a *supporting information* file

A clean copy of the edited manuscript (uploaded as the new *manuscript* file)”"

Response: A clean copy of the revised manuscript has been uploaded as the new *manuscript* file.

3. Thank you for stating in your Funding Statement:

"-KT and BD

-Partially funded by: University of Ghana Research Fund (UGRF/10/ILG-081/2016-2017) awarded to KT (main investigator) and BD (co-investigator).

-No. The funders had no role in study design, data collection and analysis, decision to publish, or preparation of the manuscript."

Please provide an amended statement that declares *all* the funding or sources of support (whether external or internal to your organization) received during this study, as detailed online in our guide for authors at http://journals.plos.org/plosone/s/submit-now.

Response: The declaration statement for the funding or sources of support received during this study has been amended, as suggested by the Editor.

Please also include the statement “There was no additional external funding received for this study.” in your updated Funding Statement.

Response: The statement ‘*There was no additional external funding received for this study*’ has been included in the updated Funding Statement in the revised manuscript.

Please include your amended Funding Statement within your cover letter. We will change the online submission form on your behalf.

Response: The amended Funding Statement has been included in the cover letter.

4. In your Data Availability statement, you have not specified where the minimal data set underlying the results described in your manuscript can be found.

Upon re-submitting your revised manuscript, please upload your study’s minimal underlying data set as either Supporting Information files or to a stable, public repository and include the relevant URLs, DOIs, or accession numbers within your revised cover letter. For a list of acceptable repositories, please see http://journals.plos.org/plosone/s/data-availability#loc-recommended-repositories. Any potentially identifying patient information must be fully anonymized.

Response: The study’s minimal underlying dataset has been uploaded as a Supporting Information file and the URL link insert in the cover letter as requested. All patient information has been fully anonymized.

We will update your Data Availability statement to reflect the information you provide in your cover letter.

Response: The authors are grateful for this kind gesture.

5. We note you have included a table to which you do not refer in the text of your manuscript. Please ensure that you refer to Table 5 in your text; if accepted, production will need this reference to link the reader to the Table.

Response: Table 5 has been referenced in the revised manuscript (Line 229- 231).

6. Please review your reference list to ensure that it is complete and correct. If you have cited papers that have been retracted, please include the rationale for doing so in the manuscript text, or remove these references and replace them with relevant current references. Any changes to the reference list should be mentioned in the rebuttal letter that accompanies your revised manuscript. If you need to cite a retracted article, indicate the article’s retracted status in the References list and also include a citation and full reference for the retraction notice.

Response: The authors have reviewed the reference list and can confirm that it is complete and correct. Moreover, none of the articles cited in the manuscript has been retracted. Furthermore, authors have also added three references to support the sensitivity and specificity of the kits used in the analysis (ref number 18 through to 20).

**Response to additional comments from the Editor:**

The authors demonstrated the prevalence of overt and occult HBV infection among patients on hemodialysis at a Tertiary Hospital in Ghana. I have some comments as follows:

1. Table 1 should present the clinical features of all patients (n=104).

Response: The initial 104 participants were tested for the presence of HBsAg. Age, gender, HBsAg-viral load was determined for all using the Elecsys from Roche Diagnostics. The structured questionnaire captured the demographic information and other clinical features, i.e. vaccination and blood transfusion status of all the patients.

Tables 1 has been edited to provide clarity on the above explanations. The Methods and Results sections have also been edited accordingly (Lines 110 – 125 and 180 – 185).

2. The titles of other tables need to show which category (HBsAg-negative or HBsAg-positive) the patients belong to.

Response: The titles of Tables 2 – 7 have been edited to indicate the category of the study patients with respect to their HBsAg status.

3. The patient number of this study was relatively small. It should be described as a limitation.

Response: The relatively small sample size has been indicated as a limitation at the conclusion section of the revised manuscript (Lines 334 – 337).

**Response to comments from Reviewer #1:**

1. Given the disproportionate risk of HBV infection among hemodialysis patients, the authors should address the availability of national policies or guidelines for prevention and control of hepatitis B among general and at-risk populations including hemodialysis patients. Further to this, the authors do not address the burden of chronic hepatitis B or hepatitis C or HBV-HCV co-infection in Ghana. Neither do they address the national hepatitis B vaccination program which are all relevant in providing context for this study (Introduction section).

Response: Our focus is mainly on HBV infection among hemodialysis patients. We did not look at HCV or HBV-HCV co-infection because of our focus. This suggestion is welcomed and can be considered in our follow-up comprehensive study which will factor in other infectious agents. The need for relevant policies to reduce or prevent HBV infection has been suggested in the conclusion section (lines 330 -334)

2. For all assays used, please provide the sensitivity / assay cut-off values / lower limit of detection where relevant (Method section).

Response: This has been included in the method section (lines 128-129; 136-137, 139-140; and 155-156)

3. The sampling and the data are old (between September 17, 2017 and December 21, 2017). The current information on prevalence of HBV infection has been surely changed (Method section).

Response: The data presented in this manuscript were obtained from samples collected approximately 6 years ago. Even though the authors agree that the current prevalence of HBV infection might have changed, we are convinced that the data in this manuscript is a vital reference for subsequent studies on HBV infection in Ghana.

4. This study is not controlled. The findings from the hemodialysis patient group is not compared with that from the control group. None of the tables provided in this manuscript provide data on the control population. Please add the demographic, clinical and study findings of the control group, this significantly impacts on the interpretation of the overall findings (Result section).

Response: The authors agree with the reviewer that data for a control (non-haemodialysis) group were not included in the study design. We are also conscious that inclusion of a control group will significantly impact the interpretation of the data. Nonetheless, the relevant statistical analysis were performed for the data illustrated in Tables 4, 5 and 7. Moreover, suitable groups were used as reference for the estimation of odds ratio in Table 5. Hence, the authors are of the opinion that the dataset in the manuscript are in support of the overall objectives of the study. Furthermore, this is a hospital-based cross-sectional study.

**Response to comments from Reviewer #2:**

Authors should be able to distinguish between false OBI, patients who might be in the reactivation phase of occult hepatitis, and those who are truly positive for HBsAg but could not be detected by the ELISA system. Indeed, during the reactivation phase of OBI, HBsAg is sometimes negative in the first weeks.

Response: In true OBI cases, HBV DNA in serum/plasma is intermittent, and when detectable, it is usually lower than 200 IU/mL. In fact, HBV-DNA levels were below 200 IU/ml in all our cases.

“False OBI” should be considered when serum HBV DNA levels are comparable to those usually detected in cases of overt HBV infection. This did not occur in any of our cases.

The diagnosis of HBV reactivation in OBI patients can be made in the case of (a) HBsAg reappearance and/or at least 1 log increase above the LLOD of serum HBV DNA in a subject with previously undetectable HBsAg and serum HBV DNA and (b) at least 1 log increase of serum HBV DNA in a subject with previously detectable HBV DNA [Raimondo , Locarnini S, Pollicino T et al. Taormina Workshop on Occult HBV Infection Faculty Members. Update of the statements on biology and clinical impact of occult hepatitis B virus infection. J Hepatol 2019; 71: 397–408]. Our study was cross-sectional, so we could not evaluate this possibility.
